# Dispersion Behaviour of Silica Nanoparticles in Biological Media and Its Influence on Cellular Uptake

**DOI:** 10.1371/journal.pone.0141593

**Published:** 2015-10-30

**Authors:** Blanka Halamoda-Kenzaoui, Mara Ceridono, Pascal Colpo, Andrea Valsesia, Patricia Urbán, Isaac Ojea-Jiménez, Sabrina Gioria, Douglas Gilliland, François Rossi, Agnieszka Kinsner-Ovaskainen

**Affiliations:** Nanobiosciences Unit, Institute for Health and Consumer Protection, European Commission Joint Research Centre (JRC), via E. Fermi 2749, 21027 Ispra, (VA), Italy; Brandeis University, UNITED STATES

## Abstract

Given the increasing variety of manufactured nanomaterials, suitable, robust, standardized *in vitro* screening methods are needed to study the mechanisms by which they can interact with biological systems. The *in vitro* evaluation of interactions of nanoparticles (NPs) with living cells is challenging due to the complex behaviour of NPs, which may involve dissolution, aggregation, sedimentation and formation of a protein corona. These variable parameters have an influence on the surface properties and the stability of NPs in the biological environment and therefore also on the interaction of NPs with cells. We present here a study using 30 nm and 80 nm fluorescently-labelled silicon dioxide NPs (Rubipy-SiO_2_ NPs) to evaluate the NPs dispersion behaviour up to 48 hours in two different cellular media either supplemented with 10% of serum or in serum-free conditions. Size-dependent differences in dispersion behaviour were observed and the influence of the living cells on NPs stability and deposition was determined. Using flow cytometry and fluorescence microscopy techniques we studied the kinetics of the cellular uptake of Rubipy-SiO_2_ NPs by A549 and CaCo-2 cells and we found a correlation between the NPs characteristics in cell media and the amount of cellular uptake. Our results emphasize how relevant and important it is to evaluate and to monitor the size and agglomeration state of nanoparticles in the biological medium, in order to interpret correctly the results of the *in vitro* toxicological assays.

## Introduction

Nanotechnological products are attracting increasing interest in biomedicine and industry as they offer novel solutions for a variety of applications [1,2]. Despite a huge number of *in vitro* and *in vivo* studies aiming to evaluate the risk associated with these formulations, limited progress has been achieved in this domain. NPs characterization data are often not sufficient or not accurate enough to allow proper comparison of results [3,4]. Due to their small size, NPs possess unique properties compared with bulk material since their enhanced surface area to mass ratio results in greater chemical and biological reactivity. Surface properties including electric charge and hydrophobicity are crucial for the dispersion characteristics and each modification of size, shape, surface coating and charge can lead to modified interactions with biological structures and consequently can alter the cell response [5–9]. It is particularly important is to characterize accurately the properties of NPs in the relevant biological environment to understand and interpret correctly the results of an *in vitro* study. The proteins present in the cellular medium and cellular components can interact with NPs and form a protein corona on their surface, leading to modified surface properties and subsequently influencing the cytotoxicity and cellular internalization [10–12]. In some situations, interaction with proteins can lead to the destabilization of colloidal systems favoring the formation of agglomerates. This has been shown to be an important factor in determining cellular response to NPs including pro-inflammatory reactions, generation of oxidative stress and genotoxicity [13–16]. Moreover, the size of agglomerates has to be considered, since it has been shown that nano-sized agglomerates may be less easily internalized by the cells than either monodisperse NPs or larger agglomerates [17]. A careful characterization of the properties of NPs in the biological medium and in contact with cells is also needed for understanding the effect of the complex biological environment on the NPs dispersion and on the effective dose of NPs reaching the cell layer. This latter point has been addressed in many dosimetry studies [18,19], and enables the impact of NPs deposition on the living cells to be determined. Indeed, the increased size of agglomerates could promote gravitational sedimentation and lead to a locally higher concentration of NPs on the surface of cells that are usually placed on the bottom of the wells for *in vitro* studies [20]. However, the behaviour of the agglomerates is more complex and their sedimentation velocity depends not only on their size but also on their density and diffusion characteristics [21].

In this study we focused on the assessment of the characteristics of NPs in the cell culture environment and on understanding the influence of these characteristics on the observed biological effect, in particular on the cell uptake of NPs. Amorphous fluorescent Rubipy-SiO_2_ NPs of two different sizes, 30 nm and 80 nm, were selected for the study, since fluorescent labelling allows rapid and direct detection of NPs in cells using easily accessible techniques. We employed two human cell lines frequently used in nanotoxicity studies: adenocarcinoma alveolar basal epithelial cells (A549) [22], and colorectal adenocarcinoma cells (CaCo-2) [23,24]. Characterization of NPs including size distribution, zeta potential and fluorescence parameters was carried out both in PBS and in all tested cell culture media taking into account the time-frame of the cell experiments and the specific conditions such as temperature or humidity. The deposition of NPs on the well bottom was measured in different media using a fluorescence spectrophotometer. Finally the cell uptake was evaluated in complete medium containing 10% of serum and in serum-free medium using flow cytometry and fluorescence microscopy. As a result, a correlation of the physico-chemical properties of SiO_2_ NPs dispersion with the observed biological response was observed.

## Materials and Methods

### Nanoparticles

To obtain monodisperse fluorescent particles of silicon dioxide with sizes in the range from 20–110 nm a synthesis method was developed based on an adaptation of a procedure previously described by Kitaev et al. [25], where a water/amino acid (arginine or lysine) mixture is placed in a glass vessel and a smaller volume of an immiscible organic liquid (cyclohexane) is added and allowed to float above the aqueous phase. This bi-phase solution is then heated (if necessary) and allowed to stabilize at the desired reaction temperature with slow stirring of the aqueous layer. At this point TEOS is gently added to the organic phase where it preferentially dissolves. The system is then left under slow stirring for time periods of a few hours to several days. During this time there is a slow diffusion of TEOS from the upper organic layer into the aqueous phase where hydrolysis and condensation occurs with the nucleation and growth of small SiO_2_ particles with a narrow size distribution.

In this study the same basic method was adopted except that the aqueous phase containing the arginine or lysine catalyst was doped with 15 mg/100 ml of a cationic inorganic fluorescent dye: Ruthenium-tris(2,2′-bipyridyl) dichloride (Ru(II)(bypy)_3_). The cationic nature of the fluorescent dye results in its rapid incorporation into the SiO_2_ particles as they grow. This method was found to be able to produce particles with different sizes in the range 20–110 nm by appropriate selection of the catalyst (arginine) concentration, reaction time and reaction temperature. For the preparation of 30 nm particles the reaction was conducted at 20°C for 24 hours using an arginine concentration of 136 mg/100 ml. For the preparation of 80 nm particles the reaction was conducted at 20°C for 72 hours using an arginine concentration of 45 mg/100 ml.

Before the biological experiments Rubipy-SiO_2_ NPs were purified in centrifugal filters (Amicon Ultra, 10 K, Milipore, Italy) at 3000 rpm for 10 min at room temperature and suspended in sterile PBS at the required concentration (30 nm) or two times higher concentration (80 nm). The fluorescence spectrum of the purified Rubipy-SiO_2_ NPs was checked and compared with the original suspension for the calculation of the concentration.

### Cell lines and culture conditions

A549 (human epithelial lung cells) and CaCo-2 (human epithelial colorectal adenocarcinoma cells) were obtained from European Collection of Cell Cultures (Sigma-Aldrich, Italy). A549 cells were cultured under standard cell culture conditions (37°C, 5% of CO_2_, 90% of humidity) in F-12 Nutrient Mixture (Ham) medium containing 10% of heat inactivated Newborn Calf Serum (New Zealand origin), 1% penicillin–streptomycin and 5 mM HEPES buffer. CaCo-2 cells were cultured under standard cell culture conditions in Dulbecco’s modified Eagle’s medium with high glucose (4.5 g/L), supplemented with 10% of heat inactivated Foetal Bovine Serum (North American origin), 1% penicillin-streptomycin, 4 mM L-glutamine and 1% non-essential amino acids. All cell culture reagents were purchased from Invitrogen (Invitrogen, Italy). The experiments were performed between passages 1–10 after defrosting of cells from liquid nitrogen.

### Physicochemical characterization of Rubipy-SiO_2_ NPs

The size distribution of Rubipy-SiO_2_ NPs was determined by centrifugal liquid sedimentation (CLS) in a sucrose density gradient using a CLS Disc Centrifuge model DC24000UHR (CPS Instruments Europe, Netherlands) and by Dynamic Light Scattering (DLS) using a Zetasizer Nano ZS instrument (Malvern Instruments, UK). The samples were prepared as a suspension of NPs at 1 mg/ml in water or in cell culture media (CCM) appropriate for the corresponding cell line, either containing 10% serum (complete CCM) or serum-free. In addition, cell pre-conditioned serum-free medium was prepared by incubation with sub-confluent A549 or CaCo-2 cells for the corresponding time-point. Before the CLS measurements the NPs suspensions were incubated at 37°C in the conditions of the cell uptake study: 1 to 48 hours incubation in complete CCM or 1 to 5 hours incubation in serum-free cell pre-conditioned medium. To evaluate the effect of cell debris and large molecules on the NPs dispersion stability the cell pre-conditioned medium was filtered through the centrifugal filters (Amicon Ultra 10 K) at 3000 rpm for 10 min in room temperature, then the measurements were repeated. The zeta potential of the NPs in water, PBS and cell culture media for both A549 and CaCo-2 cells was determined by electrophoretic mobility (Zetasizer Nano-ZS instrument, Malvern Instruments, UK). In order to obtain the fluorescence spectra of Rubipy-SiO_2_ NPs the stock solutions of 30 and 80 nm NPs were diluted either in water, in PBS or in complete cell culture medium at concentrations of 0,2 mg/ml and the fluorescence emission intensity was measured using a fluorescence spectrophotometer (Cary Eclipse, Varian, Australia Pty Ltd).

### Analysis of uptake of SiO_2_ NPs by flow cytometry

The cells were seeded in 12-well plate (Costar) at a density of 100 000 cells/well, grown until 80–90% confluence and exposed to 100–200 μg/ml of 30 nm or 80 nm Rubipy-SiO_2_ NPs in fresh complete or serum-free medium. After the end of the exposure the cells were washed 3 times in PBS, trypsinised and blocked with a complete cell culture medium, washed again in PBS and analysed immediately by flow cytometry.

Evaluation of cell associated fluorescence, forward scattering (FSC) and side scattering (SSC) were carried out using a CyFlow space flow cytometer (Partec, Munster, Germany) and the data were analysed using FCS Express 4 software (De Novo, Los Angeles, CA). Laser excitation was at 488 nm and the emission band pass wavelength was 590/50 nm (FL-2) for Rubipy-SiO_2_ NPs related fluorescence. A minimum of 10 000 cells per sample were analysed; cells debris, nanoparticles and doublets were excluded from the analysis by gating on the FSC versus SSC log graph and on the FL-2 area versus FL-2 width graph respectively. The results are reported as median of the cell associated fluorescence intensity averaged between three independent experiments (2 replicas each) and after the subtraction of cell autofluorescence.

### Fluorescence microscopy

A549 and CaCo-2 cells were seeded at a density of 8x10^4^ cells/well on 4-chamber polystyrene vessel tissue culture-treated glass slides (BD Falcon, Italy). 24 h after seeding, cells were exposed to 200 μg/ml of 30 or 80 nm Rubipy-SiO_2_ NPs for different exposure times at 37°C either in complete cell culture medium containing 10% serum or in serum-free medium. Following exposure, cells were washed 3 times in PBS to remove unbound particles, fixed with 4% (v/v) paraformaldehyde in PBS and permeabilised with 0.1% (v/v) Triton X-100 in PBS (Sigma-Aldrich, Italy). To delimit cell boundaries and discriminate between NPs present inside or outside of the cell actin filaments were stained for 40 min at room temperature with AlexaFluor 488-conjugated Phalloidin (Invitrogen, Italy) diluted 1:100 in PBS. The nuclei were counterstained with Hoechst 33342 dye (Dako, Italy), diluted 1:2000 in PBS. After staining, the cells were washed in PBS and mounted for microscopy. Images were acquired with an Axiovert 200 M inverted microscope equipped with ApoTome slide module and Axiovision 4.8 software (Carl Zeiss; Jena, Germany), using a 40×/1.0 objective lens.

### Kinetics of NPs deposition

A549 cells were seeded in 12-well plate (Costar) at a density of 100 000 cells/well, grown until 80–90% confluent and exposed to 100 μg/ml (30 nm) or 200 μg/ml (80 nm) Rubipy-SiO_2_ NPs in fresh complete or serum-free medium. A separate plate was prepared for each time point (10 min, 30 min, 1 h, 3 h and 5 h) including the wells with non-treated cells, cells exposed to Rubipy-SiO_2_ NPs, and control wells with Rubipy-SiO_2_ NPs dispersion, in triplicates. After a given exposure time the supernatant from the wells with the exposed cells (deposition wells) was transferred to the wells with non-treated cells (supernatant wells) and the fluorescence intensity was measured in all wells, excitation/emission 495/590 nm, by a multiwell plate reader (FluoStar, Omega, BMG Labtech, Offenburg, Germany). The signals from non-treated cells were used as blanks and subtracted from the readings; the signal from the control wells with Rubipy-SiO_2_ NPs dispersion was treated as 100% and used to calculate a percentage of the NPs in deposition wells and in the supernatant. In parallel, the same experiment was performed in the absence of cells, in order to compare the kinetics of NPs deposition in both conditions.

## Results

### Size measurement and colloidal stability of Rubipy-SiO_2_ NPs in cellular media

The size distribution of Rubipy-SiO_2_ NPs was measured by DLS and CLS first in water and in serum-free medium suitable for A549 and CaCo-2 cell cultures (Table 1). The obtained hydrodynamic size values measured by DLS were: 30–33 nm for 30 nm SiO_2_ NPs and 78–80 nm for 80 nm SiO_2_ NPs, whereas the CLS values were 26 nm and 62–68 nm, respectively. The dispersions of both sizes of Rubipy-SiO_2_ NPs in water and in serum-free media were stable at least up to 72 h. The electric charge was strongly negative in water (-37 mV and– 52 mV for respectively 30 and 80 nm NPs) and less negative in PBS and cell culture media (-14 mV and -16 mV) (S1 Table).

**Table 1:** Particle size distribution in water and serum-free cell culture media.

		DLS		CLS
		(Z-average)	PDI	(by weight)
SiO_2_ 30	in H_2_0	30.2 ± 4	0.052	26.1 ± 11
	in A549 CCM	32.8 ± 5	0.081	26 ± 8
	in CaCo-2 CCM	33.1 ± 5	0.075	26 ± 10
SiO_2_ 80	in H_2_0	80.2 ± 9	0.044	68.8 ± 22
	in A549 CCM	80.3 ± 9	0.003	62 ± 16
	in CaCo-2 CCM	78.8 ± 9	0.023	61.8 ± 16

**Fig 1:**
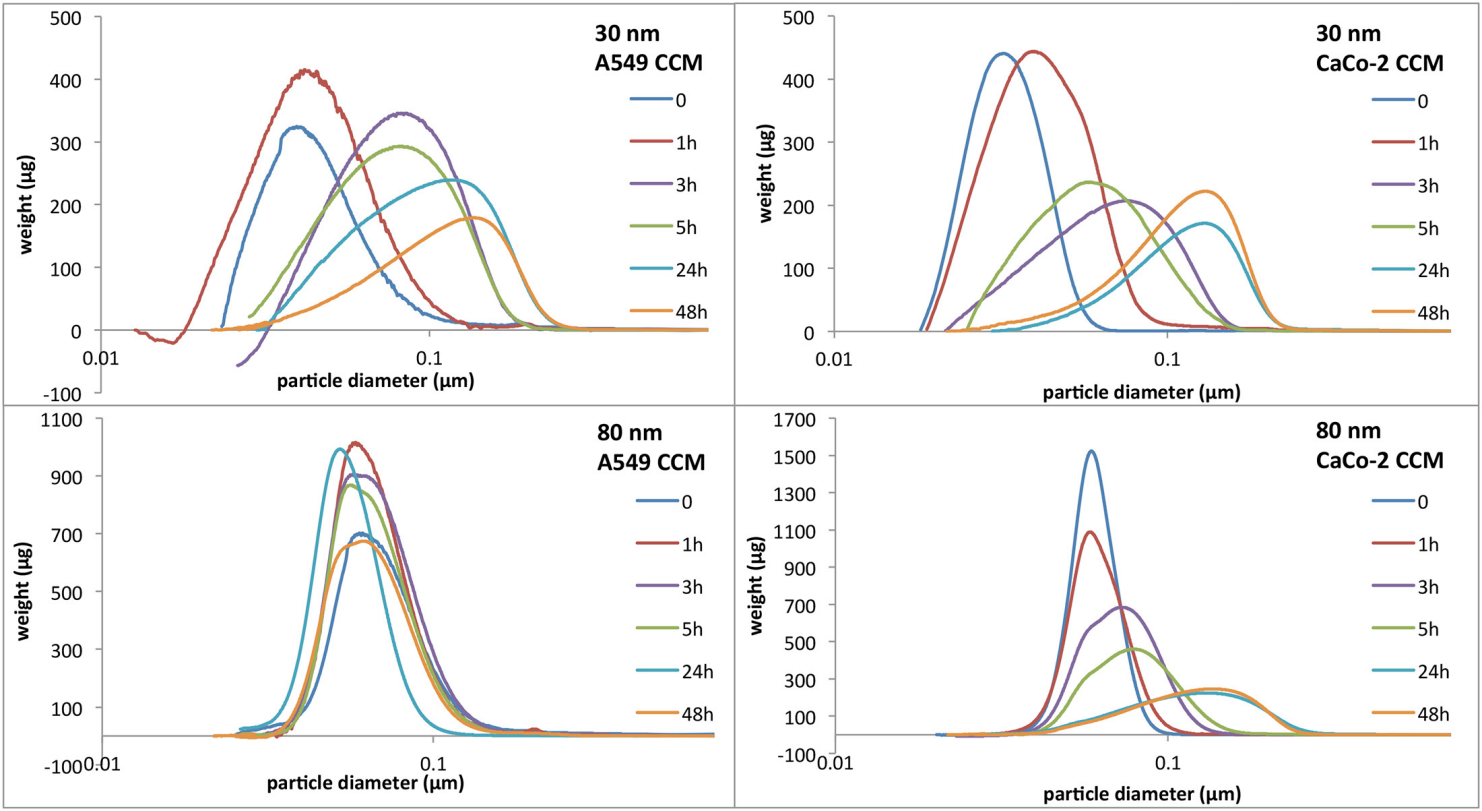
Size distribution of Rubipy-SiO_2_ NPs in complete media. Rubipy-SiO_2_ NPs were suspended either in A549 (left) or CaCo-2 (right) complete cell culture medium (10% of serum) at concentration 1 mg/ml and the size distribution was measured in time up to 48 hours by CLS.

When suspended in complete culture media in the presence of 10% serum, both sizes of Rubipy-SiO_2_ NPs showed clear differences in the dispersion behaviour (Fig 1). The average diameter of 30 nm Rubipy-SiO_2_ NPs measured by CLS increased after 24 h in both media to around 130 nm and the size distribution shifted to larger values in a time-dependent manner clearly indicating agglomeration of NPs. The number of particles in the agglomerate could be estimated by using the formula of Sterling [26]. The size of an agglomerate of 130 nm corresponds to roughly 30 NPs by assuming a spherical agglomerate with fractal dimension of 2.5 and a packing factor of 0.637 [19,27]. The density can be determined leading to a value of 1.6 g/cm^3^, instead of 2.3 g/cm^3^ which is the density of bulk silica. Using these parameters allowed us to use the theoretical model of the particles deposition in the well (S1 Fig) and comparing these values with the experimental findings.

The dispersion of 80 nm SiO_2_ NPs was different in two different cell culture media: in A549 complete medium the dispersion remained stable for the whole duration of the experiment (48 hours) whereas in CaCo-2 complete medium the 80 nm NPs behaved similarly to the 30 nm particles, showing an increased polydispersity, an average size of 130 nm after 24 h and agglomeration (Fig 1). In this case, the size of the agglomerate corresponds to only a few NPs (<5) leading to a density of roughly 1.8 g/cm^3^.

To confirm our CLS results we performed transmission electron microscopy (TEM) analysis of the NPs suspended in cell culture media, both serum-free and complete. We employed a sample preparation procedure that minimizes agglomeration artefacts in dried TEM samples and allows a more homogenous distribution of the particles on the TEM grid.[28] In serum-free A549 and CaCo-2 medium we observed more or less a uniform layer of NPs surrounded by traces of dried culture medium (S2 Fig), which is indicative of a good dispersion in solution. In complete medium, particularly in CaCo-2 complete medium, the abundant presence of serum proteins together with other components was a limiting factor that hampered the proper visualization and analysis of NPs morphology and dispersion. Nevertheless, we could distinguish the agglomerates of NPs from the covering precipitates of salts and proteins. Only in one condition (80 nm NPs in A549 complete medium) the NPs were distributed more homogenously among the whole TEM grid, even if embedded in the protein mixture, indicating absence of agglomeration. This is in agreement with the CLS results (Fig 1).

**Fig 2:**
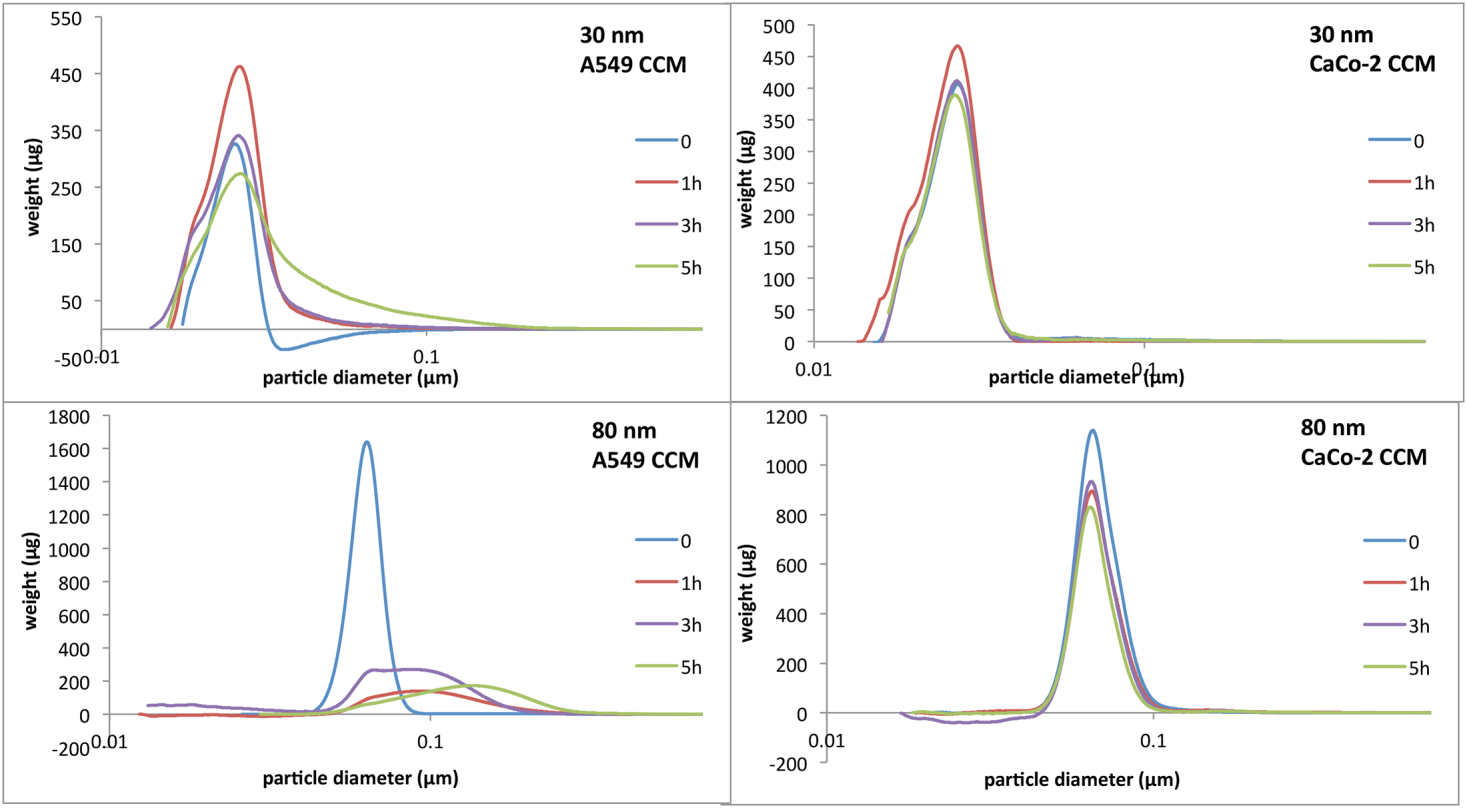
Effect of cell presence on NPs size characteristics. Rubipy-SiO_2_ NPs 30 and 80 nm were suspended in cell culture medium without serum and pre-conditioned by incubation with cells for 1, 3 and 5 hours and the size distribution was measured by CLS.

**Fig 3:**
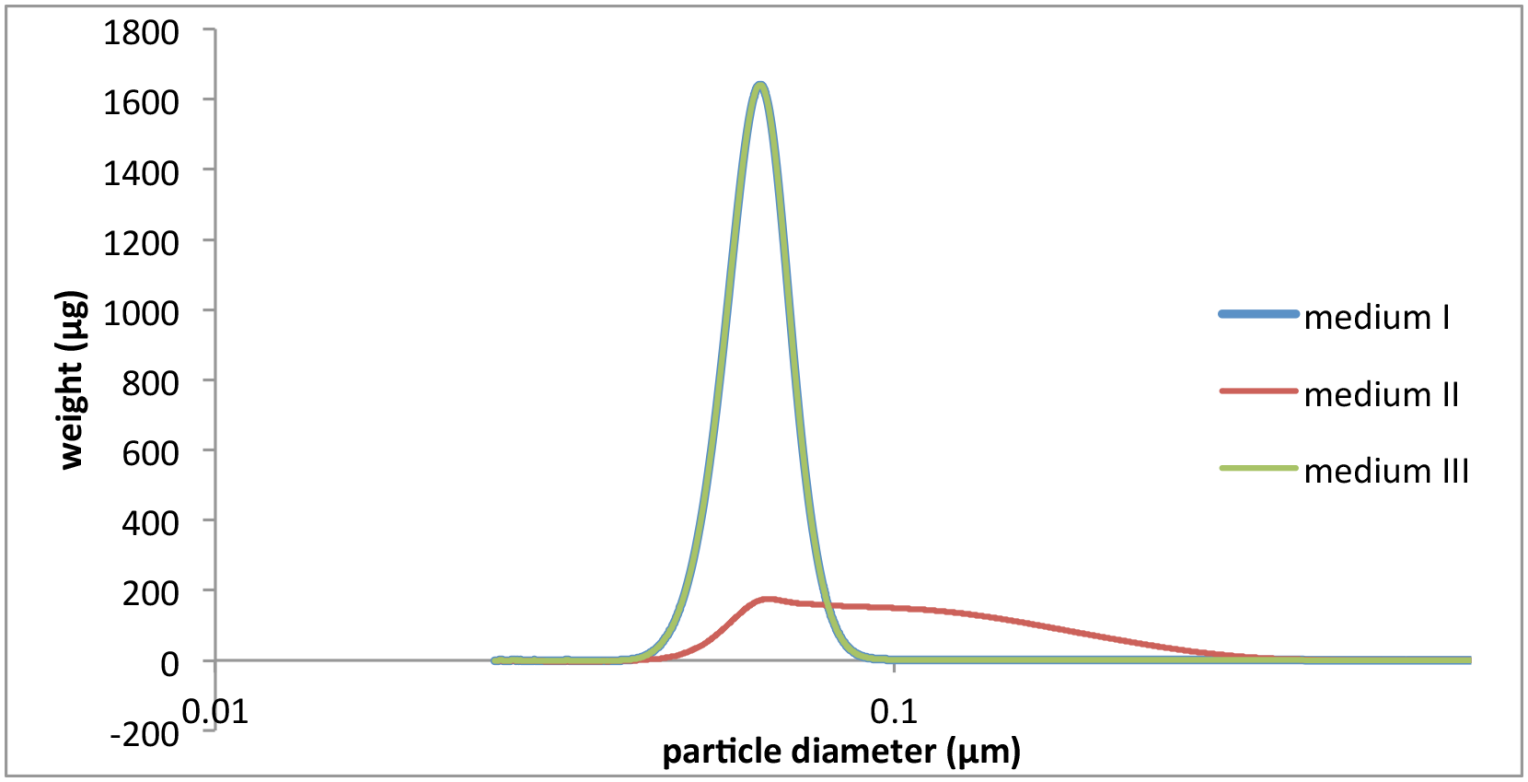
Effect of cell presence on NPs size characteristics. CLS size measurement of 80 nm Rubipy-SiO_2_ NPs suspended at 1 mg/ml in a) serum free A549 medium (med I), b) serum free A549 medium pre-conditioned by the incubation with cells (med II), c) filtrated medium II (med III), after 24 h incubation at 37°C.

In addition, we assessed the effect of cell presence on NPs dispersion characteristics. The fresh serum-free medium was pre-incubated with sub-confluent cells for 1, 3 or 5 hours and then was used to prepare Rubipy-SiO_2_ NPs dispersions. After 1, 3 or 5 hours incubation of NPs in cell pre-conditioned media at 37°C the size distribution was evaluated by CLS (Fig 2). 30 nm SiO_2_ NPs remained stable in these conditions and the size did not differ from the size in fresh medium, whereas 80 nm Rubipy-SiO_2_ NPs showed the presence of agglomerates already after 1 hour incubation in A549 cell pre-conditioned medium, but not in CaCo-2 pre-conditioned medium. On SEM images of 80 nm Rubipy-SiO_2_ NPs in A549 cell pre-conditioned medium (S3 Fig right) we can observe regular round particles of variable sizes, mostly much bigger than the ones dispersed in water (S3 Fig left); these are agglomerates consisting probably of 2 or more NPs and proteins. Furthermore, the dispersion of 80 nm Rubipy-SiO_2_ NPs in the same cell pre-conditioned medium but centrifugally filtered showed the same size distribution as in fresh serum-free medium (Fig 3), indicating that the observed agglomeration was induced by the cell debris or large proteins secreted by the cells.

In order to verify if the modified colloidal stability of NPs in different cell media conditions can be explained by the variations in the protein corona composition we isolated, washed and separated the proteins bound to NPs by SDS-polyacrylamide gel electrophoresis (SDS-PAGE). The results of SDS-PAGE indicated that, indeed, slightly different bands were visible in two different A549 and CaCo-2 complete media conditions as well as in serum-free cell pre-conditioned media (S4 Fig). However, little difference, if any, could be observed comparing 30 nm and 80 nm NPs protein corona in the same conditions. Further studies on this are currently ongoing.

### Fluorescence characteristics of Rubipy-SiO_2_ NPs

Fluorescence spectra were determined for Rubipy-SiO_2_ 30 and 80 nm NPs. The fluorescence intensity of Rubipy-SiO_2_ 80 nm NPs was higher than that of the 30 nm NPs at the same mass concentration (S5 Fig). Furthermore the fluorescence emission peak was detected at 603 nm for Rubipy-SiO_2_ 80 nm and at 597 nm for Rubipy-SiO_2_ 30 nm. The observed blue shift probably originated from the difference in dye concentration in the two nanoparticle types [29]. The fluorescence spectra of Rubipy-SiO_2_ NPs suspended in complete media were recorded over several hours and compared with the spectra of NPs suspended in water. The fluorescence signal was similar in both conditions and stable over the time, indicating that NPs agglomeration and protein corona formation had no effect on the fluorescence intensity (S5 Fig).

### Determination of cytotoxicity

In our study no cytotoxic effects were observed after 72 h exposure of cells to Rubipy-SiO_2_ NPs of either 30 nm or 80 nm in complete cell culture medium, as assessed by the MTT assay (S6 Fig and not shown). In serum-free conditions already after 3 h exposure to 30 nm Rubipy-SiO_2_ NPs (and slightly also for 80 nm Rubipy-SiO_2_ NPs) a morphological change and a decrease of cell size were observed by fluorescent microscopy. These morphological effects were not visible when cells were incubated in serum-free medium in the absence of NPs suggesting that they were due to NPs exposure (S7 Fig). However, the metabolic activity of cells assessed by MTT assay was significantly decreased only after 48 h exposure to 30 nm Rubipy-SiO_2_ (S6 Fig), showing that the cell metabolism was less affected by the exposure. Nevertheless, because of the observed cytotoxicity, the concentration of 30 nm Rubipy-SiO_2_ NPs applied for the study of the kinetics of cell uptake was decreased to 100 μg/ml instead of 200 μg/ml and the exposure time reduced to a maximum of 5 h in serum-free conditions.

### Kinetics of cell uptake of Rubipy-SiO_2_ NPs

**Fig 4:**
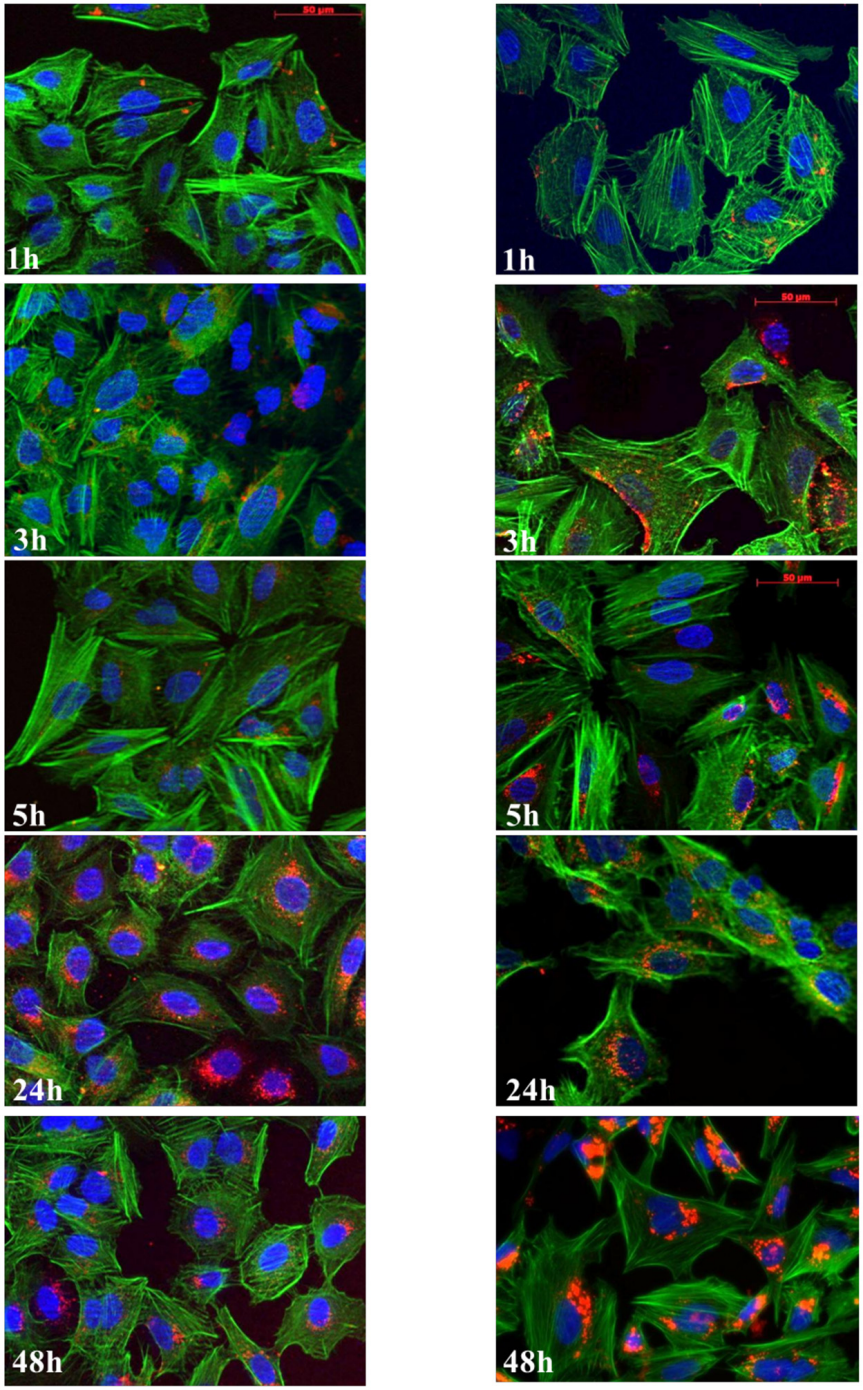
Kinetics of the cell uptake of Rubipy-SiO_2_ NPs. A549 cells were exposed to 200 μg/ml of 30 nm (left column) and 80 nm (right column) Rubipy-SiO_2_ NPs in complete CCM for different time intervals up to 48 h, and stained with Hoechst-33342 (nuclei; blue) and with AlexaFluor 488-conjugated phalloidin (actin; green), and observed by fluorescence microscopy (Rubipy-SiO_2_ NPs, red). Scale bar: 50 μm.

Cellular uptake of Rubipy-SiO_2_ was studied qualitatively by fluorescence microscopy (allowing visualization of NPs inside the cells) and quantitatively by flow cytometry. Fluorescence microscopy studies showed that both 30 and 80 nm-sized Rubipy-SiO_2_ NPs were efficiently and in a time-dependent manner taken up by A549 cells and that they accumulated progressively in the perinuclear area, but not in the cell nucleus (Fig 4). The orthogonal view of z-stack images of cells exposed to Rubipy-SiO_2_ NPs provided evidence that NPs were localized within the cell boundaries indicating that fluorescence of the exposed cells measured by flow cytometry was mainly due to the internalized NPs and not NPs attached to the cell membrane (S8 Fig).

**Fig 5:**
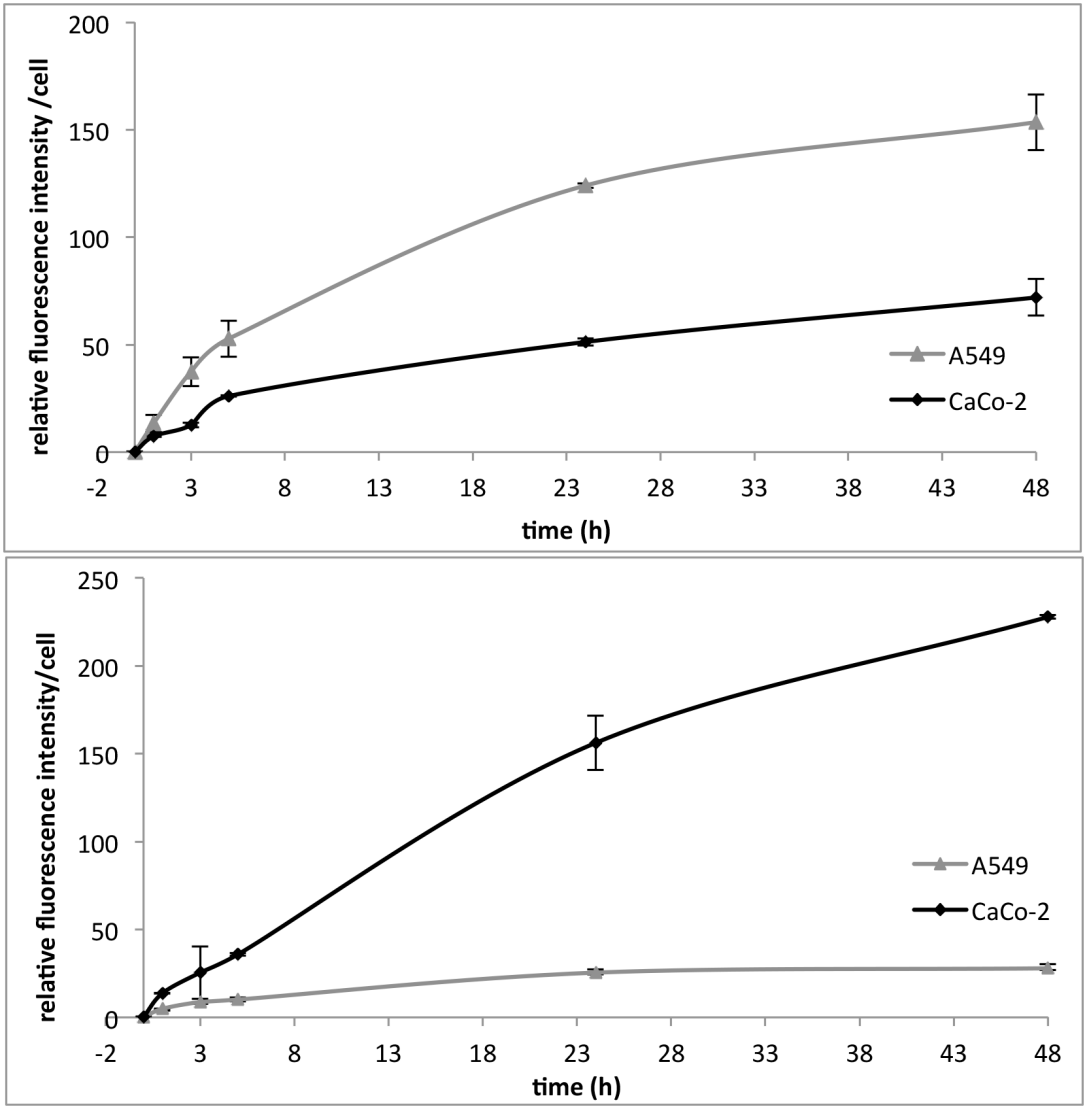
Quantification of the cell uptake of Rubipy-SiO_2_ NPs. The cellular uptake of Rubipy-SiO_2_ NPs, was quantified by flow cytometry after exposure of A549 and CaCo-2 cells to 100 μg/ml of 30 nm Rubipy-SiO_2_ NPs (up) or to 200 μg/ml of 80 nm Rubipy-SiO_2_ NPs (down) in complete CCM during 1 h, 3 h, 5 h, 24 h and 48 h.

The flow cytometry experiments indicated a fast cell uptake of Rubipy-SiO_2_ NPs during the first hours of exposure that slowed or ceased completely after 24 h (Fig 5). Differences in the level of the internalization were observed between the two cell lines. In complete medium, a higher uptake of 30 nm Rubipy-SiO_2_ NPs was observed in A549 cells as compared to Caco-2 cells (Fig 5, upper graph). In contrast, the 80 nm Rubipy-SiO_2_ NPs were taken up more efficiently by Caco-2 cells (Fig 5, lower graph).

**Fig 6:**
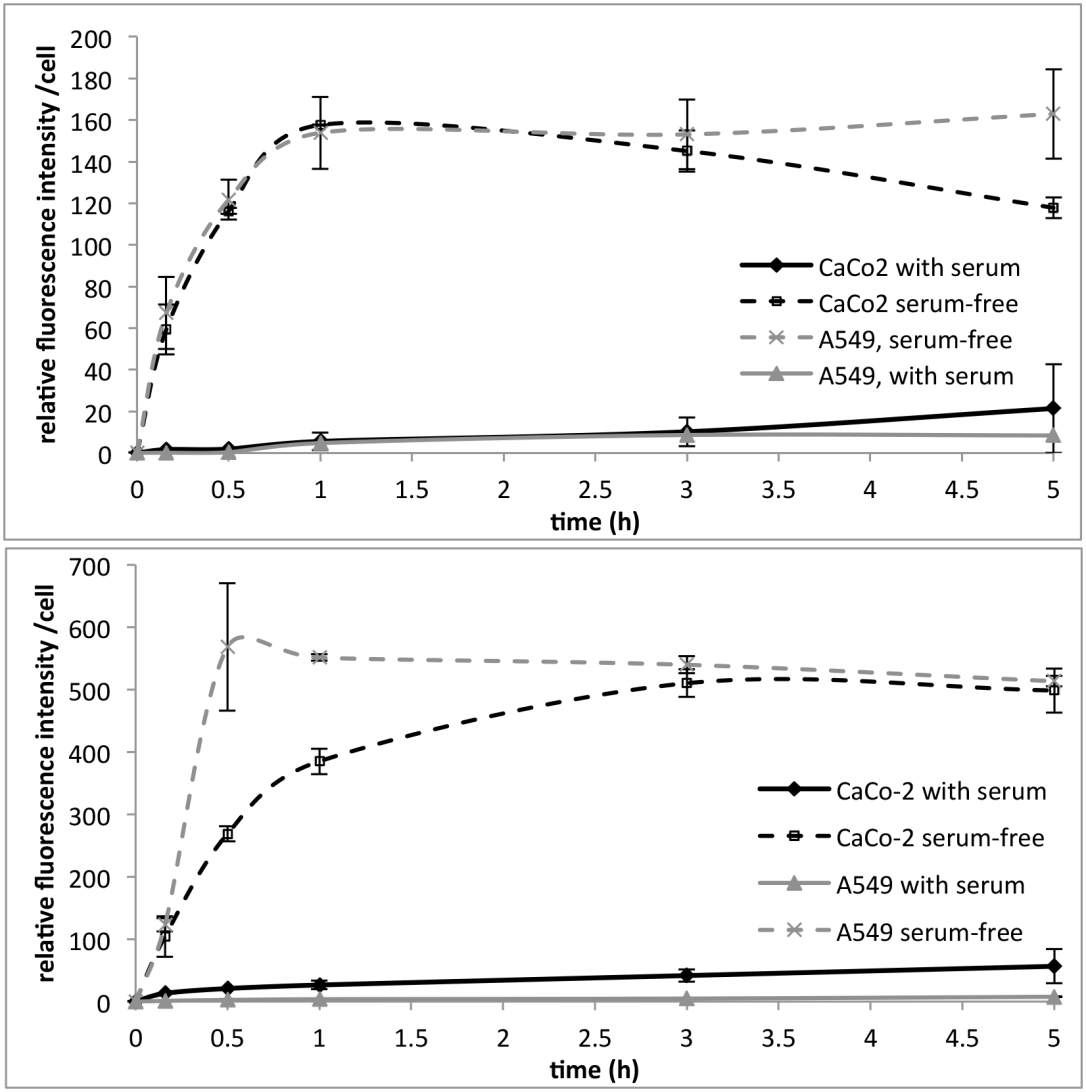
Effect of serum depletion on the kinetics of cell uptake. A549 cells and CaCo-2 cells were exposed to 100 μg/ml of 30 nm Rubipy-SiO_2_ NPs (top) or to 200 μg/ml of 80 nm Rubipy-SiO_2_ NPs (bottom) either in serum-free conditions or in complete medium. Cell associated fluorescence was quantified by flow cytometry after 10 min, 30 min, 1 h, 3 h and 5 h exposure.

In serum-free conditions the cell uptake of both sizes of Rubipy-SiO_2_ NPs much faster and to a higher level than in complete cell medium (Fig 6), showing signs of saturation already after 1 h exposure. The 80 nm Rubipy-SiO_2_ NPs were taken up faster by A549 cells as compared to CaCo-2 cells, while no significant difference in the rate of uptake was seen for the 30 nm NPs in both cell lines.

### Kinetics of NPs deposition

**Fig 7:**
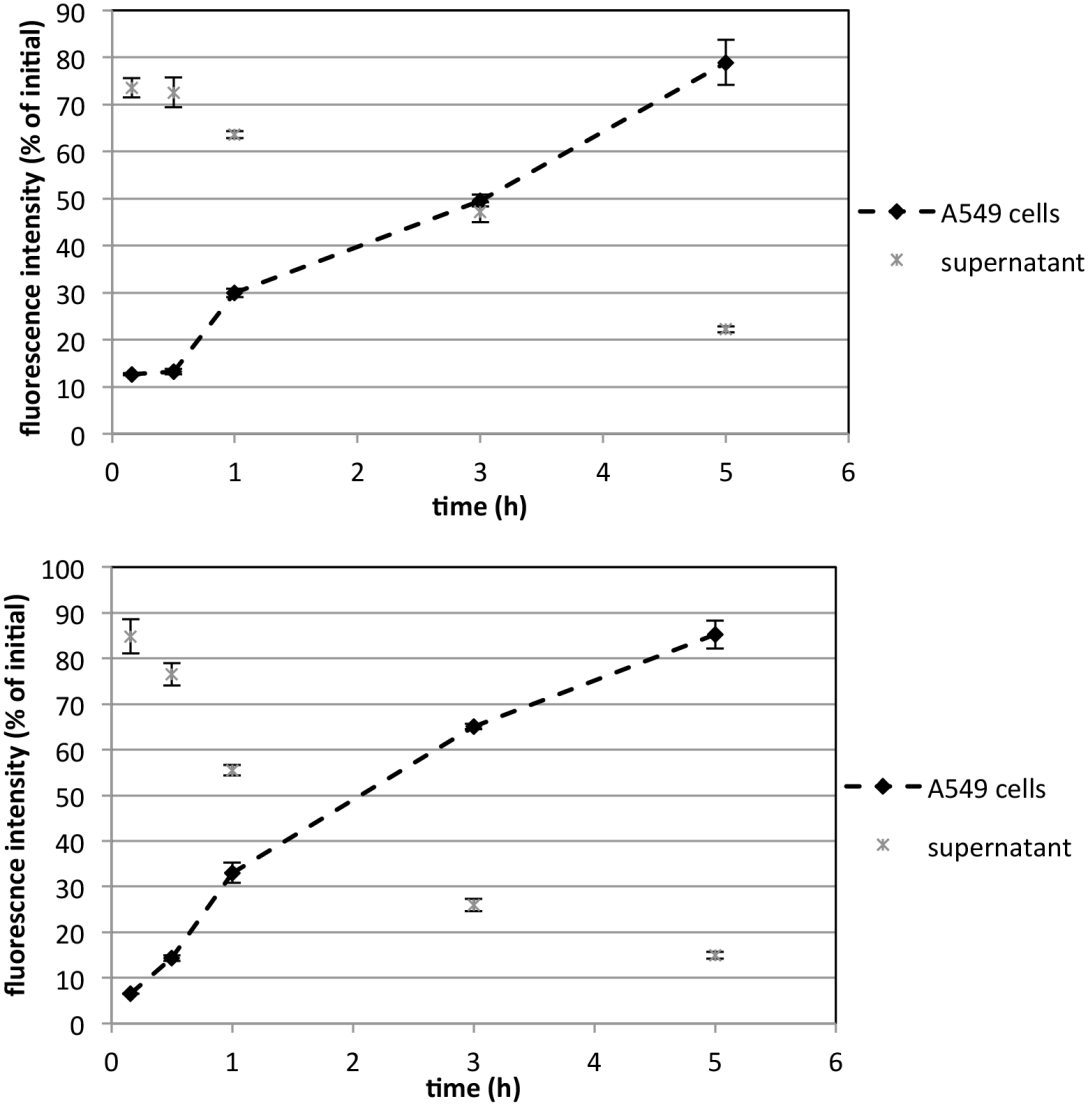
Kinetics of NPs deposition. A549 cells were incubated with 30 nm (top) and 80 nm (bottom) Rubipy-SiO_2_ NPs in serum-free conditions for up to 5 h. Fluorescence intensity of the supernatant and of the cell layer was measured with a spectrophotometer at every time point.

In parallel to the cell uptake studies, and using the same conditions, the kinetics of deposition of silica NPs at the cell surface or at the cell-free (plastic) surface was evaluated by quantification of fluorescence intensity in the multiwell plate reader. In the study performed in complete medium both in the presence and in the absence of cells only a small percentage of the NPs (30 nm and 80 nm) was detected on the bottom of the well (deposition part) and most of them were found in the supernatant part over the duration of the study, i.e. up to 48 h (data not shown). Similar results were obtained in serum-free medium in the absence of cells. However, in the study performed on A549 cells in serum-free conditions, the fluorescence of the cell layer increased continuously with time reaching 80–90% of the initial NPs suspension after 5 h. A corresponding decrease was observed in the supernatant part (Fig 7). The kinetics of the deposition was similar for both sizes of Rubipy-SiO_2_ NPs.

## Discussion

The complex behaviour of NPs in cell culture media should always be considered in nanoparticle-cell interaction studies *in vitro*. Several processes such as dissolution, aggregation, sedimentation and formation of a protein corona have an important influence on the surface properties and on the stability of NPs in the biological environment, and consequently on the NPs interaction with cells. The aim of the present study was to investigate the differences in the dispersion behaviour of silica NPs of two different sizes in two distinct cell culture media in the presence or absence of 10% of serum, and to evaluate how the varying NPs characteristics in the different *in vitro* conditions affect the NPs intracellular uptake and cytotoxicity.

It is worth stressing that the characterization of NPs dispersion in standard conditions for the *in vitro* biological experiments, i.e. in serum-containing cell culture medium, remains a challenge despite the recent improvement of the analytical methods [30]. The presence of the proteins is a limiting factor for a correct interpretation of the obtained data. Furthermore, the concentration of NPs in the sample should be ideally equal to the one used for the biological experiments, so relatively low. Very often this is not possible since in order to obtain good quality data a sufficiently high sample concentration and a high particle to protein ratio is required. Therefore, the choice of appropriate methods for assessing size distribution in cell culture medium is challenging. In this study, the size of Rubipy-SiO_2_ NPs in complete medium containing 10% serum was measured only by CLS, as DLS data are not reliable for polydisperse samples or when agglomerates are present [21,31]. CLS was shown to be one the most accurate methods to evaluate the size of NPs and their agglomeration state, especially in cell culture media [32], but care has to be taken in the interpretation of the data. CLS measures the sedimentation time of a complex under centrifugation which can then be related to its mass and size. The time of sedimentation is a function of the fixed experimental parameters such as viscosity of the fluid, density of the gradient of sucrose, the rotational speed and the dimension of the disc and two unknowns parameters related to the NPs, namely the NPs density and diameter. However, the particle density usually is assumed to be the density of the bulk material (2.3 g/cm^3^ for silica) leading to an underestimation of the NPs diameter. This is due to the fact that the bulk density does not account for contribution of the hydration layer covering the NPs which decreases the nanoparticle apparent density. Indeed in our case the NPs diameter values obtained from CLS were calculated by using the density of bulk silica so are slightly underestimated and lower as compared to the DLS values. Nevertheless, despite the method limitations, the CLS spectra provided important information on the behaviour of the particles in complete medium and showed clear differences in the dispersion behaviour of both sizes of Rubipy-SiO_2_ NPs in two distinct cell culture media for both A549 and Caco-2 cells. The changes in NPs size distribution and their agglomeration were time-dependent, highlighting that NPs dispersion behaviour should be monitored in the biological conditions throughout the whole experiment and not only one time-point.

Another important point is the influence of living cells on the NPs stability and deposition. Indeed, when exposed to medium without serum, that has been pre-conditioned with the A549 cells, 80 nm NPs agglomerated already after 1 hour, while the same NPs were stable when suspended in serum-free medium that has not been in contact with cells as well as in medium with 10% of serum. Pre-conditioning of medium with the CaCo-2 cells did not affect the size distribution of 80 nm NPs. Moreover, NPs did not agglomerate if the A549 pre-conditioned medium was filtered before adding the nanoparticles. This observation highlights the importance of the source and type of the proteins present on NPs surface, since depending on their origin (serum, cell secreted proteins) and properties they can either stabilize or destabilize NPs dispersion and consequently influence nanoparticle-cell interactions [33]. However, it is likely that this will vary depending on NPs composition, since another study demonstrated the opposite trend for gold NPs: an increased NPs agglomeration in the filtrate of A549 pre-conditioned medium comparing to non-filtrated pre-conditioned medium [34]. Moreover, depending on NPs size and surface curvature, and despite the same surface chemistry, different types of proteins contribute to protein corona formation, and lead to different behaviour of the dispersion in the same medium [35,36]. Here, in agreement with existing reports [34], we observed an increased tendency to agglomerate for the smaller NPs than for the larger NPs, even if the profile of the protein corona was very similar for both sizes of Rubipy-SiO_2_ NPs.

We also evaluated if changes in size distribution of silica NPs have an effect on their cytotoxic potential. The size of the NPs has been highlighted in many studies as one of the main parameters determining particle cytotoxicity [37,38]. Smaller NPs were reported to be more toxic than the larger ones, inducing oxidative stress and inflammatory process, whereas the agglomeration of NPs could modulate the cellular response and decrease associated cytotoxicity [15,39]. In our experiments, both sizes of Rubipy-SiO_2_ NPs were not toxic to cells in complete cellular medium, though some cytotoxicity of 30 nm NPs was detected in serum-free conditions. Moreover, changes in cell morphology and decrease in cell size were observed after exposure to Rubipy-SiO_2_ NPs in serum-free conditions by fluorescent microscopy. This observation was already reported by other researchers [33,40], and is most probably a result of the increased vulnerability of the cells depleted from serum and/or increased adhesion of NPs to the cell membrane in these conditions [41] enhancing nanoparticle-cell interaction.

To evaluate further whether there is a correlation between NPs characteristics in cell media and the NPs cell interaction in the corresponding conditions, we determined the kinetics of cellular uptake of Rubipy-SiO_2_ NPs in A549 and CaCo-2 cells using flow cytometry and fluorescence microscopy techniques. Clear differences in the level of the internalization were observed between the two cell lines, but several parameters need to be considered in order to correctly interpret the results. Cellular uptake of NPs is a function of the amount of NPs reaching the cell monolayer (the dose) and of the uptake kinetics parameters such as adsorption rate, desorption rate, and density of binding sites [42]. The amount of NPs reaching the cell monolayer depends on the physical properties of the NPs (size, mass density) and their stability in culture medium (state of agglomeration). The kinetics parameters are related to cell types, physical chemical properties of the NPs and the nature of the protein corona. The results of characterization of the NPs in culture medium show that the particles tend to agglomerate forming objects of different sizes depending on the cell medium, cell type and time of exposure. NPs of 30 nm in both complete culture media created agglomerates of a similar size and density, suggesting that in both situations the dose of 30 nm NPs reaching the cells monolayers was approximately the same. Thus, the higher uptake observed for A549 cells was due most likely to a higher affinity of NPs to these cells and to the cell phenotype. CaCo-2 cells are smaller than A549 cells, proliferate more rapidly and are able to achieve higher cell density in the wells leading to lower NPs content per cell. On the other hand, 80 nm NPs remained monodispersed in A549 complete medium and agglomerated slightly in CaCo-2 complete medium, which should result, according to a theoretical dosimetry model (S1 Fig), in a slightly higher dose reaching CaCo-2 cell layer than A549 cells. However, the difference in the dose does not explain completely the difference in the level of uptake, suggesting higher affinity of 80 nm NPs to CaCo-2 cells. As we expected, the protein corona profile was different in A549 complete medium and in CaCo-2 complete medium, and this was shown to be an important parameter determining NPs uptake [43]. Additionally, in agreement with existing reports [33,44,45] the cell uptake of both sizes of Rubipy-SiO_2_ NPs was, in the absence of serum, much faster than in complete cell medium, confirming that the serum protein corona on the nanoparticles modulates the NPs cellular uptake.

In parallel to the cell uptake study we verified if and how NPs are deposited and adsorbed at the cell surface (or at the plastic surface in cell-free experiments) and if these findings were reflected in the observed cellular uptake. In the complete medium only a small amount of the NPs (both 30 nm and 80 nm) was found in the deposition part, as the level of the cellular internalization was in these conditions rather low and difficult to detect by multiwell plate reader. Moreover, all other NPs were coated by the proteins and did not adhere to the cells surface or to the plastic, thus were removed from the deposition wells and remained in the supernatant part during the measurement. On the contrary, in serum-free A549 medium the deposition of NPs was fast and almost complete after only 5 h, exceeding the estimation calculated in the dosimetry model (S1 Fig) which is based solely on the particle size and density and does not consider the cellular component. We can then conclude that the reason for the enhanced cellular uptake observed in serum-free conditions comparing to complete medium was the increased dose of NPs reaching the cell layer in these conditions. Importantly, the kinetics of the deposition was similar for both sizes of Rubipy-SiO_2_ NPs: 30 nm, which were stable in these conditions maintaining their original size and 80 nm, which were strongly agglomerating. Regarding the kinetics of the cell uptake quantified by flow cytometry, we can hypothesize that the deposition of NPs continued also after the cell uptake was saturated. Interestingly, this tendency was not observed in the absence of cells, where only few percent of the initial NPs in suspension was deposited and attached to the plastic surface. We can then conclude that the cellular component was critical in determining the deposition of the NPs in the well.

## Conclusions

In this study we compared the size distribution and the stability of the dispersion of two sizes (30 and 80 nm) of amorphous Rubipy-SiO_2_ NPs in two different cellular media with and without 10% of serum. Both types of silica NPs had the same surface chemistry and only differed in size, yet they behaved differently in cellular media containing the proteins, either originating from added serum or secreted by the cells. By a careful selection of methods we were able to assess the NPs dispersion characteristics in conditions similar to the uptake study and to correlate these findings with the results of the *in vitro* experiments. We observed particularly low cellular uptake in the conditions where NPs dispersion was stable for the whole duration of the experiment. The depletion of serum during the exposure to NPs led to enhanced NPs deposition on the cell layer resulting in increased cell uptake and cytotoxicity. Moreover we showed that the presence of living cells can not only change the size distribution and the colloidal stability of NPs but also strongly influences the kinetics of the NPs deposition, independently of their state of agglomeration. Therefore characterization studies of NPs in the biological media and the dosimetry models should consider the cellular component as well as time-dependent and cell-dependent changes in the extracellular environment.

## Supporting Information

S1 Fig Model of the deposition of Rubipy-SiO_2_ NPs. (PDF)

S2 Fig Dispersion of Rubipy-SiO_2_ NPs in cell culture media. (PDF)

S3 Fig Effect of cell pre-conditioning of medium on NPs size. (PDF)

S4 Fig Components of protein corona bound to Rubipy-SiO_2_ NPs in different cell culture conditions. (PDF)

S5 Fig Fluorescence spectra of Rubipy-SiO_2_ NPs suspended in cell culture medium. (PDF)

S6 Fig Metabolic activity of cells exposed to Rubipy-SiO_2_ NPs. (PDF)

S7 Fig Effect of serum depletion on cell morphology. (PDF)

S8 Fig Internalization of Rubipy-SiO_2_ NPs by A549 cells. (PDF)

S1 Materials and Methods (PDF)

S1 Table Zeta-potential values of Rubipy-SiO_2_ NPs. (PDF)
