# Supplementary material for: Dispersion Behaviour of Silica Nanoparticles in Biological Media and Its Influence on Cellular Uptake
Source: PLoS One. 2015 Oct 30;10(10):e0141593. doi: 10.1371/journal.pone.0141593 (PMC4627765; doi:10.1371/journal.pone.0141593)
Supplement: S1 Fig — (PDF) [file pone.0141593.s001.pdf]

S1 Fig. Model of the deposition of Rubipy-SiO_2 NPs. Fraction of SiO_2 deposited (F(t)) NPs in reference to the initial amount added to the well, assuming 100 % uptake efficiency.

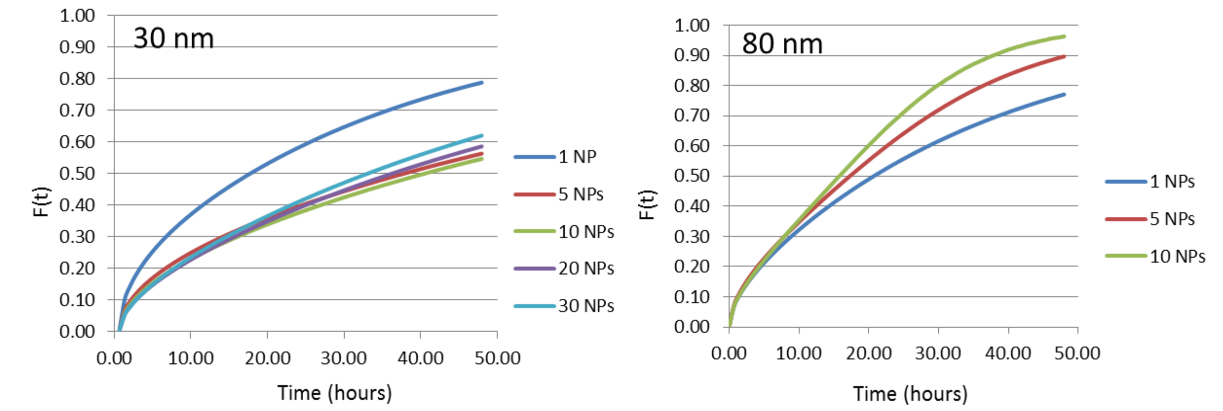

Nb of NPs	Diameter	Density
5	68	1.79
10	90	1.69
20	119	1.6
30	110	1.55

Nb of NPs	Diameter	Density
5	182	1.79
10	240	1.69
