# Supplementary material for: Dispersion Behaviour of Silica Nanoparticles in Biological Media and Its Influence on Cellular Uptake
Source: PLoS One. 2015 Oct 30;10(10):e0141593. doi: 10.1371/journal.pone.0141593 (PMC4627765; doi:10.1371/journal.pone.0141593)

**S2 Fig. Dispersion of Rubipy-$SiO_2$ NPs in cell culture media.** Rubipy-$SiO_2$ NPs 30 nm (A) and 80 nm (B) were suspended at 1 mg/ml in A549 and CaCo-2 cell culture media either serum-free or containing 10 % of serum. After 24 h incubation at 37°C, TEM samples were prepared and TEM observation was performed.

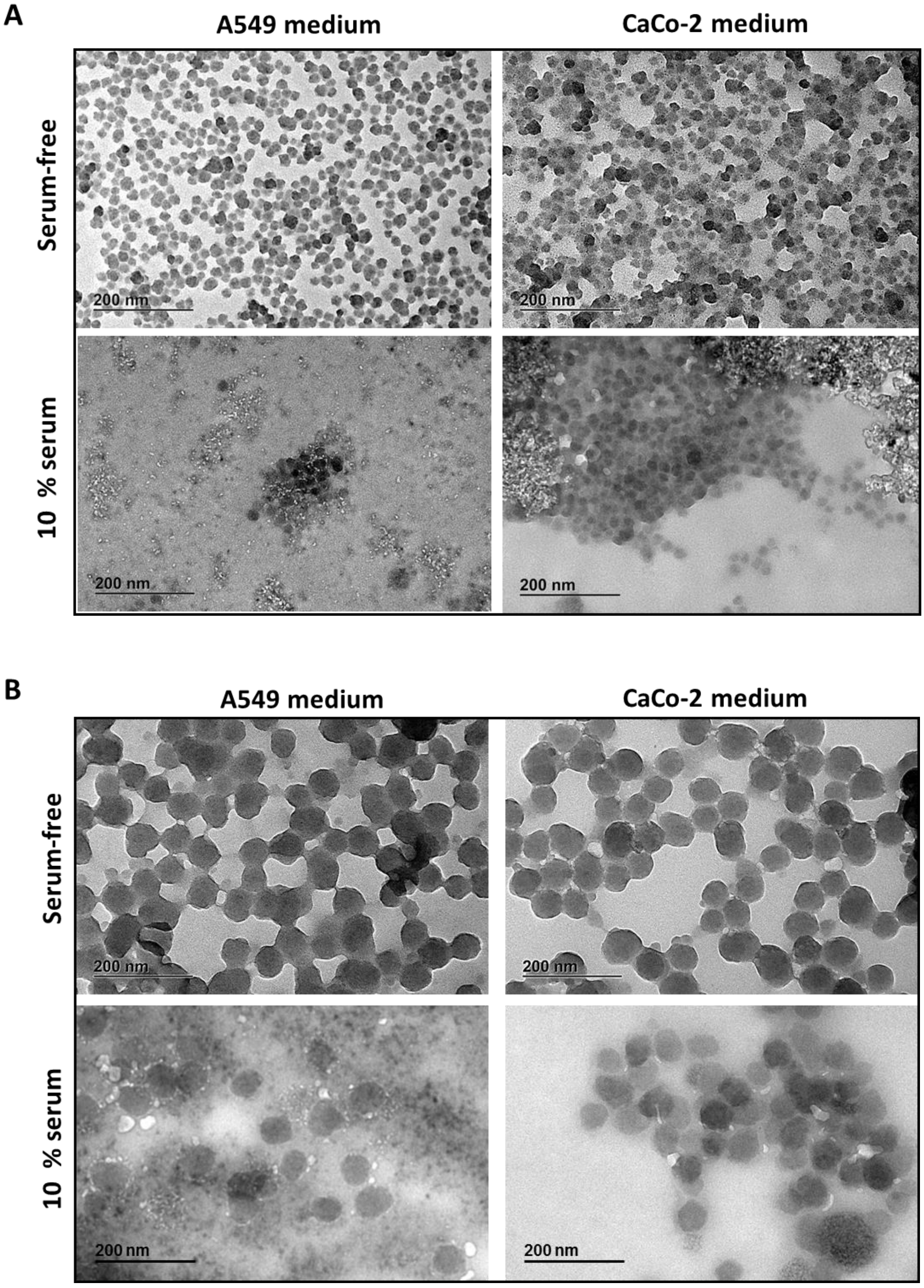

Supplement: S2 Fig — (PDF) [file pone.0141593.s002.pdf]
