# Supplementary material for: Dispersion Behaviour of Silica Nanoparticles in Biological Media and Its Influence on Cellular Uptake
Source: PLoS One. 2015 Oct 30;10(10):e0141593. doi: 10.1371/journal.pone.0141593 (PMC4627765; doi:10.1371/journal.pone.0141593)

S3 Fig. **Effect of cell pre-conditioning of medium on NPs size**. 80 nm Rubipy-SiO_2 NPs were suspended at 1 mg/ml in H_2O (left) or in cell pre-conditioned A549 medium (right) for 1 hour and the scanning electron microscopy images were prepared. The average diameter was calculated with Image J on basis of at least 100 particles. Scale bar: 2 µm.

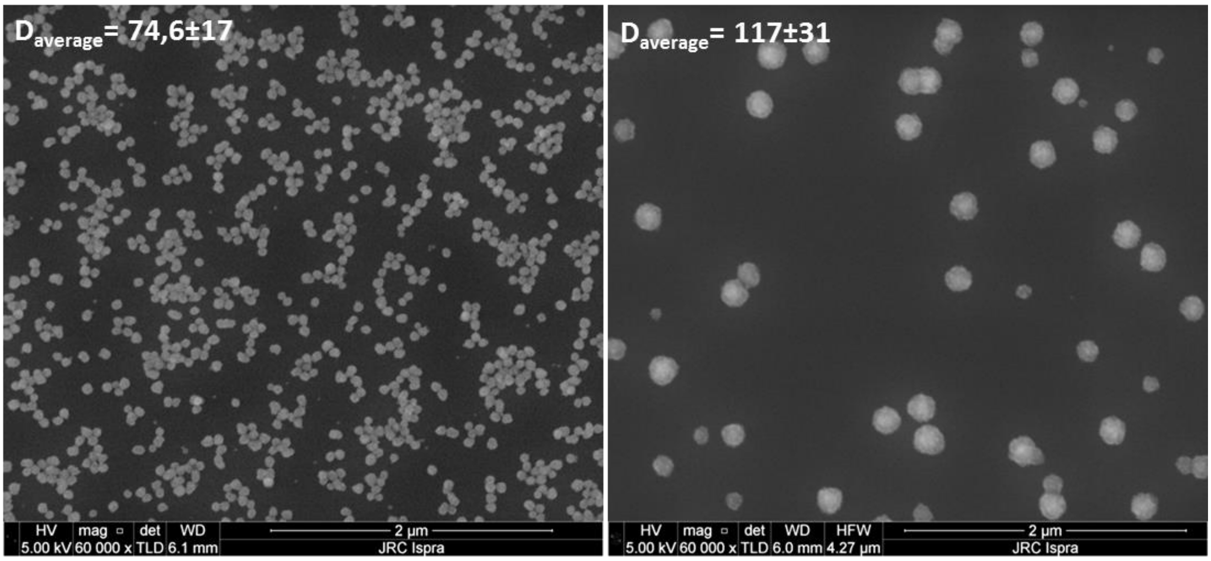

Supplement: S3 Fig — (PDF) [file pone.0141593.s003.pdf]
