# Supplementary material for: Dispersion Behaviour of Silica Nanoparticles in Biological Media and Its Influence on Cellular Uptake
Source: PLoS One. 2015 Oct 30;10(10):e0141593. doi: 10.1371/journal.pone.0141593 (PMC4627765; doi:10.1371/journal.pone.0141593)

S4 Fig. Components of protein corona bound to Rubipy-SiO_2 NPs in different cell culture conditions. Rubipy-SiO_2 NPs 30 and 80 nm were incubated in complete A549 (a) or CaCo-2 (b) medium containing 10% of serum during 24 h or in serum-free cell pre-conditioned A549 (c) or CaCo-2 (d) medium during 5 h and the proteins bound to NPs surface were washed, eluted and separated by SDS-PAGE.

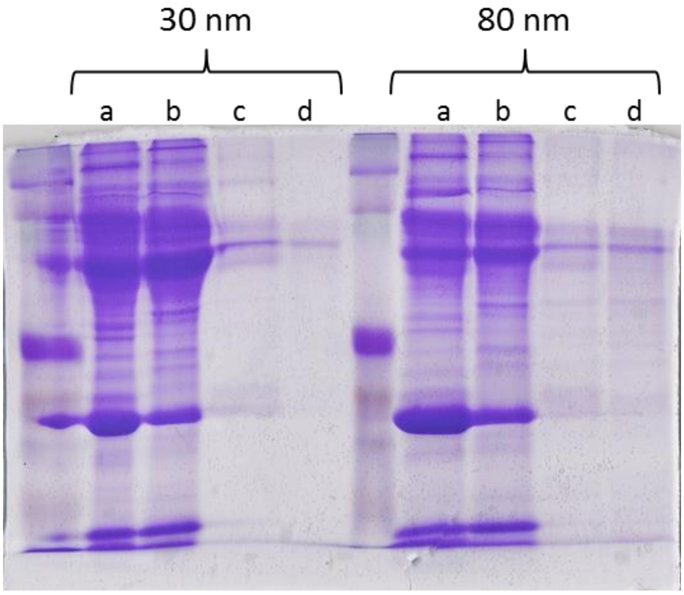

Supplement: S4 Fig — (PDF) [file pone.0141593.s004.pdf]
