# Supplementary material for: Dispersion Behaviour of Silica Nanoparticles in Biological Media and Its Influence on Cellular Uptake
Source: PLoS One. 2015 Oct 30;10(10):e0141593. doi: 10.1371/journal.pone.0141593 (PMC4627765; doi:10.1371/journal.pone.0141593)

S5 Fig. Fluorescence spectra of Rubipy-SiO_2 NPs suspended in cell culture medium. Rubipy-SiO_2 NPs 30 nm (left) or 80 nm (right) were suspended at 0,2 mg/ml in A549 complete medium (A) or in CaCo-2 complete medium (B) and the fluorescence emission was recorded immediately and after 1, 3 and 5 hours incubation at 37°C, using excitation wavelength of 460 nm.

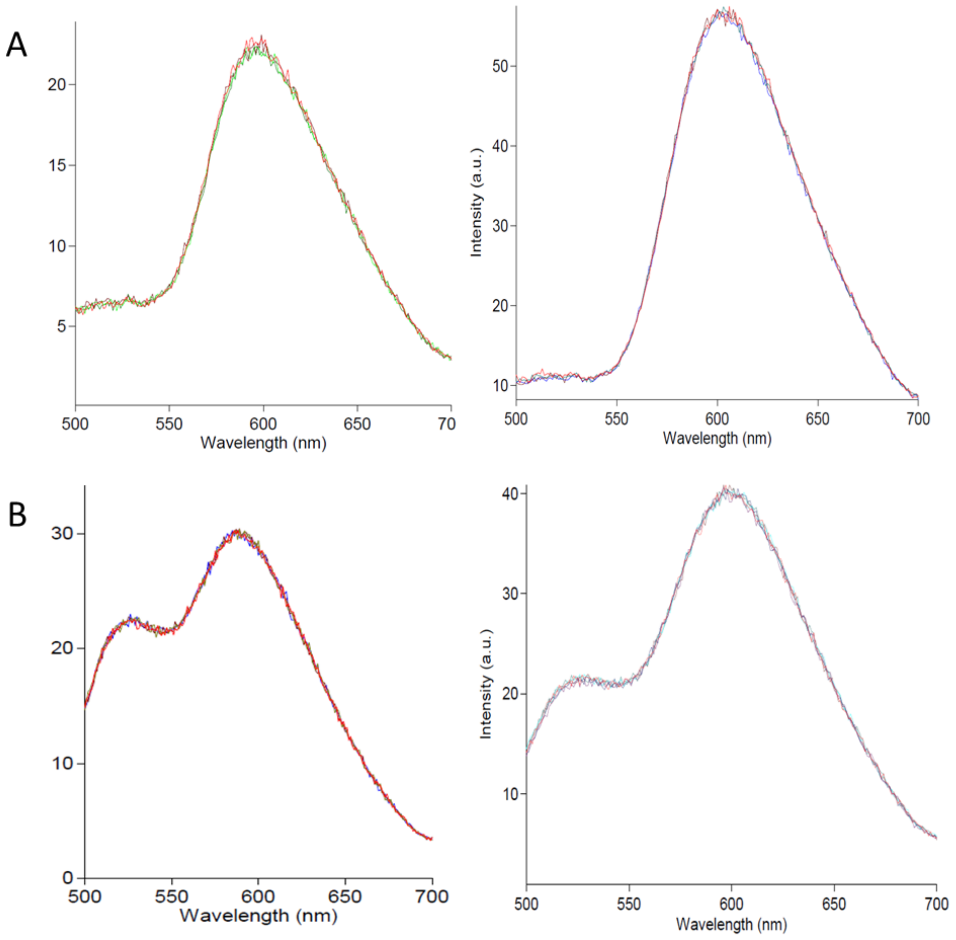

Supplement: S5 Fig — (PDF) [file pone.0141593.s005.pdf]
