# Supplementary material for: Dispersion Behaviour of Silica Nanoparticles in Biological Media and Its Influence on Cellular Uptake
Source: PLoS One. 2015 Oct 30;10(10):e0141593. doi: 10.1371/journal.pone.0141593 (PMC4627765; doi:10.1371/journal.pone.0141593)

S6 Fig. Metabolic activity of cells exposed to Rubipy-SiO_2 NPs. A549 (left) and CaCo-2 (right) cells were exposed to 30 nm Rubipy-SiO_2 NPs for 48 h in complete or serum-free medium and the MTT assay was performed.

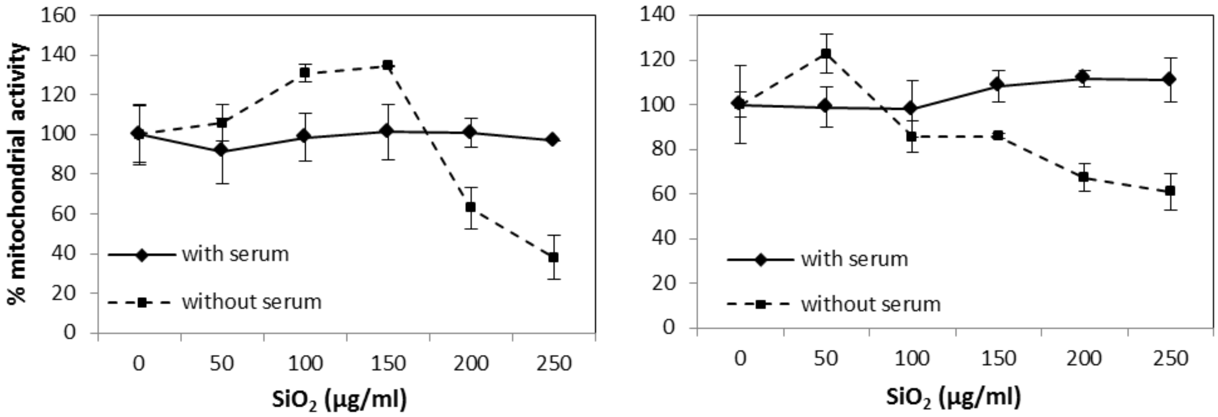

Supplement: S6 Fig — (PDF) [file pone.0141593.s006.pdf]
