# Supplementary material for: Dispersion Behaviour of Silica Nanoparticles in Biological Media and Its Influence on Cellular Uptake
Source: PLoS One. 2015 Oct 30;10(10):e0141593. doi: 10.1371/journal.pone.0141593 (PMC4627765; doi:10.1371/journal.pone.0141593)

S7 Fig. **Effect of serum depletion on cell morphology.** A549 cells were either left untreated or exposed to 200 μg/ml of 30 or 80 nm Rubipy-SiO_2NPs for 3 h at 37°C in complete cell culture medium or in serum-free medium. Actin filaments were stained with AlexaFluor 488-conjugated phalloidin (green) and nuclei were stained with Hoechst-33342 (blue). Scale bar: 20 μm.

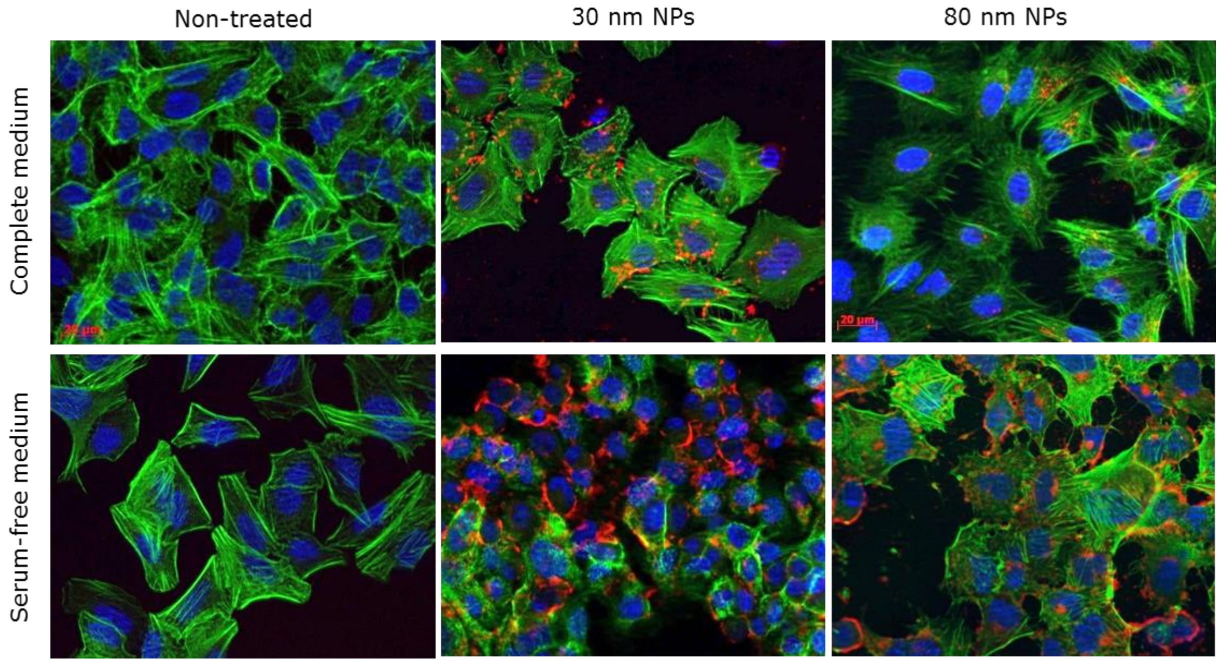

Supplement: S7 Fig — (PDF) [file pone.0141593.s007.pdf]
