# Supplementary material for: Dispersion Behaviour of Silica Nanoparticles in Biological Media and Its Influence on Cellular Uptake
Source: PLoS One. 2015 Oct 30;10(10):e0141593. doi: 10.1371/journal.pone.0141593 (PMC4627765; doi:10.1371/journal.pone.0141593)

S8 Fig. **Internalization of Rubipy-SiO_2 NPs by A549 cells.** Orthogonal view of z-stack of A549 cells was recorded after 24 h exposure to 30 nm (left) and 80 nm (right) Rubipy-SiO_2 NPs in complete CCM; nuclei were stained with Hoechst-33342 (blue), actin filaments with phalloidin (green), Rubipy-SiO_2 NPs (red). Scale bar: 20 µm.

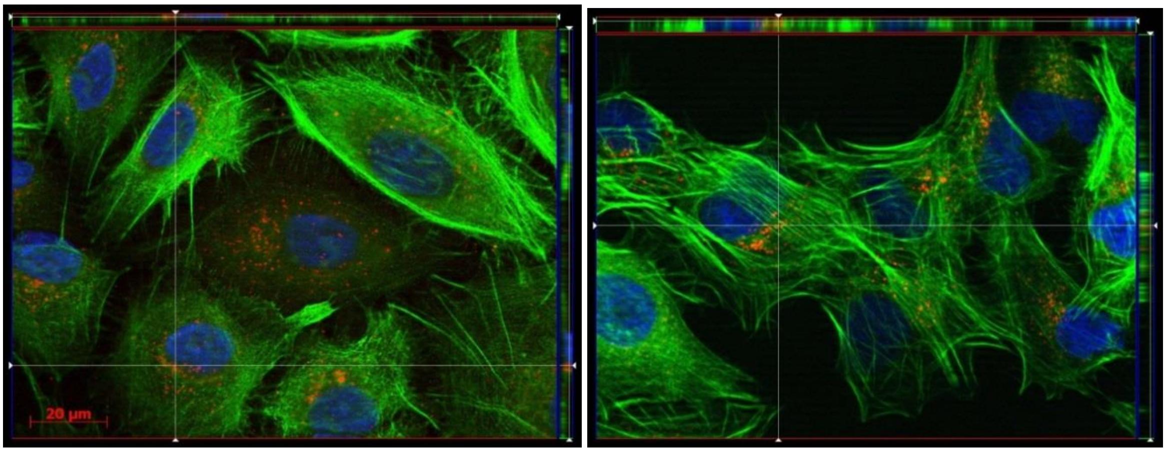

Supplement: S8 Fig — (PDF) [file pone.0141593.s008.pdf]
