# Supplementary material for: Dispersion Behaviour of Silica Nanoparticles in Biological Media and Its Influence on Cellular Uptake
Source: PLoS One. 2015 Oct 30;10(10):e0141593. doi: 10.1371/journal.pone.0141593 (PMC4627765; doi:10.1371/journal.pone.0141593)
Supplement: S1 Materials and Methods — (PDF) [file pone.0141593.s009.pdf]

Supplementary Materials and Methods

Transmission Electron Microscopy

Rubipy-SiO_2 NPs 30 nm and 80 nm were suspended at 1 mg/ml in cell culture medium, either serum-free or containing 10 % of serum, and were incubated at 37°C for 24 h.

Ultrathin Formvar-coated 200-mesh copper grids (Tedpella Inc.) were first functionalized by placing the carbon-coated side on a drop of 20 µl of Alcian blue (2 % in water) deposited on Parafilm. After 10 min incubation the grid was washed 5 times by the deposition on the drops of water placed on Parafilm and the excess fluid was removed by blotting its edge on a strip of paper tissue, leaving a rest of humidity. Finally the grid was placed on a 20 µl drop of the corresponding sample, incubated for 10 min and the excess of fluid was removed again with a paper tissue. TEM (JEOL JEM 2100, Japan) at an accelerating voltage of 200 kV was used to visualize the nanoparticles.

Scanning Electron Microscopy

A plasma polymerized poly acrylic acid thin film was deposited on a silicon wafer by means of a home-made capacitively coupled plasma reactor described elsewhere [26], at a nominal RF power of 12 W and working pressure of 20 mTorr. After deposition the film was rinsed in milli-Q water in order to remove the non-stable polymer. The thickness of the stable film was measured by means of a spectroscopic ellipsometer (VASE). Measured film thickness was 50±2 nm. Rubipy-SiO_2 NPs were suspended at 1 mg/ml in water or cell medium, then the droplets of 1 µl were manually spotted by a micropipette on poly acrylic acid thin film, let dry at room temperature and then rinsed by milli-Q water. Only strongly attached particles remained on the surface. In this way, a uniformly distributed monolayer of nanoparticles could be used for the evaluation by Scanning Electron Microscopy (SEM). SEM

measurements were performed by a FEI NOVA 600, Dual Beam, using 5 KeV acceleration voltage and acquiring secondary electrons. The average size of particles was calculated by Image J software, taking into account at least 100 particles.

Sodium dodecyl sulfate polyacrylamide gel electrophoresis (SDS-PAGE)

To investigate NPs interactions with the proteins Rubipy-SiO_2 NPs were suspended at concentration of 1 mg/ml either in complete A549 or CaCo-2 cell culture medium or in serum-free cell pre-conditioned medium and incubated at 37°C for 24 hours or 5 hours, respectively. At the end of the incubation time NPs were centrifuged for 15 min at 16 000g at 4°C and the pellets were washed 3 times with 1 ml of PBS in order to remove non-bound proteins. The supernatants were monitored for the presence of non-precipitated Rubipy-SiO_2 NPs by the fluorescence measurement. Proteins bound to NPs surface were eluted with the Laemmli sample buffer (Sigma-Aldrich, Italy), boiled at 99°C for 5 min and separated on 12% SDS-PAGE. Protein bands were visualized by Coomassie blue staining.

Evaluation of cell metabolic activity/cytotoxicity (MTT assay)

Cells were grown in 96-well cell culture plates (Costar) until 75% confluent, then exposed to silica NPs either in complete medium or in serum free medium at the concentration and time indicated, then washed in PBS. Cell viability was evaluated using the MTT [3-(4,5-dimethylthiazol-2-yl)-2,5- diphenyl-2H tetrazolium bromide] (Sigma–Aldrich) added to the cells in fresh complete culture medium at a 250 μg/ml final concentration. After 2 h of incubation at 37˚C the supernatant was removed and the precipitated formazan crystals were dissolved in 0.1 M HCl in propan-2-ol and the absorbance was quantified at 540 nm in a multiwell plate reader (FluoStar, Omega, BMG Labtech, Offenburg, Germany). In parallel, to evaluate the possibility of interference of NPs with the assay, the PBS washing containing the

silica residues from each well was transferred to empty wells, incubated with MTT reagent in the conditions of the experiment and after 2 h the absorbance at 540 nm was read in a multiwell plate reader.
