# Supplementary material for: Dispersion Behaviour of Silica Nanoparticles in Biological Media and Its Influence on Cellular Uptake
Source: PLoS One. 2015 Oct 30;10(10):e0141593. doi: 10.1371/journal.pone.0141593 (PMC4627765; doi:10.1371/journal.pone.0141593)
Supplement: S1 Table — (PDF) [file pone.0141593.s010.pdf]

S1 Table: Zeta-potential values of Rubipy-SiO_2 NPs. Rubipy-SiO_2 NPs 30 and 80 nm were suspended in MilliQ water, PBS or serum-free CCM at concentration of 1 mg/ml and the zeta-potential values (in mV) were determined by electrophoretic mobility**.**

	in H_2O	in PBS	in A549 CCM	in CaCo-2 CCM
SiO_2 30	-37.4	-14.4	-14.1	-14.7
SiO_2 80	-52.7	-16.2	-16.0	-15.5
